# Case Report: Clinical course and extended survival in a canine patient with pericardial mesothelioma managed without chemotherapeutic intervention

**DOI:** 10.3389/fvets.2026.1808005

**Published:** 2026-05-21

**Authors:** Seol-Gi Park, Seongsoo Lim, Joungsun Moon, O. I-se, Taekyu Chung, Daesik Kim

**Affiliations:** 1 Sky Institute of Veterinary Applied Science, Incheon Sky Animal Hospital, Incheon, Republic of Korea; 2 Veterinary Emergency Medicine, Department of Veterinary Clinical Science, College of Veterinary Medicine, Research Institute for Veterinary Science, Seoul National University, Seoul, Republic of Korea

**Keywords:** canine, case report, pericardial mesothelioma, pericardiectomy, pericardiocentesis, pleural effusion

## Abstract

Pericardial mesothelioma is a rare and aggressive neoplasm in dogs, typically associated with a poor prognosis and limited treatment options. While chemotherapy is commonly employed, there is limited literature describing successful management without chemotherapeutic intervention. This case report describes a 7-year-old spayed female Pomeranian presenting with acute respiratory distress caused by pericardial effusion. Initial management included pericardiocentesis and medications, but recurrence of the effusion necessitated surgical intervention. Subtotal pericardiectomy was performed, and malignant pericardial mesothelioma was confirmed histopathologically. A thoracic port was implanted postoperatively for ongoing effusion management. The patient remained clinically stable and maintained a good quality of life for 628 days without chemotherapy, supported by regular monitoring and comprehensive symptomatic care. This case highlights that prolonged survival may be achieved through aggressive effusion control and surgical intervention in dogs with pericardial mesothelioma when systemic chemotherapy is not feasible.

## 1 Introduction

Pericardial and pleural effusions constitute significant pathophysiological sequelae in canine patients with cardiovascular and pulmonary disorders, potentially precipitating life-threatening clinical manifestations if inadequately managed. Among the diverse etiologies, malignant mesothelioma represents an uncommon yet clinically significant neoplasm affecting the mesothelial lining of serosal surfaces, including the pericardium, pleura, and peritoneum. In canine patients, pericardial mesothelioma is recognized as a significant neoplastic cause of pericardial effusion, alongside hemangiosarcoma and chemodectoma (1, 2). The disease typically demonstrates an insidious progression, with clinical manifestations becoming apparent only after substantial effusion accumulation results in cardiac tamponade or respiratory compromise (3).

The etiopathogenesis of mesothelioma in canines remains poorly understood. While asbestos exposure represents a well-established risk factor for mesothelioma in human patients, a definitive causal relationship has not been established in veterinary medicine (4, 5). Diagnosis is often challenging, as conventional imaging modalities such as thoracic X-rays, echocardiography, and computed tomography (CT) often miss discrete mass lesions (6, 7). CT imaging may demonstrate pleural nodularity, pericardial thickening, or intrathoracic lymphadenopathy, potentially supporting a diagnosis of mesothelioma (1, 7, 8). Cytologic and histopathologic evaluations remain critical for confirmation (7, 9). Cytological analysis of pericardial or pleural fluid is often diagnostically limited, as it is inherently difficult to distinguish reactive mesothelial cells from neoplastic mesothelial cells based on morphological criteria alone. Histopathological examination remains essential for definitive diagnosis, with stromal invasion serving as the most reliable criterion for distinguishing malignant mesothelioma from reactive mesothelial hyperplasia (7, 10, 11).

Therapeutic management of pericardial mesothelioma is predominantly palliative, aiming to alleviate symptoms and improve quality of life. Subtotal pericardiectomy is commonly performed to prevent recurrent cardiac tamponade, providing short-term clinical benefit (6). Systemic chemotherapy protocols, including carboplatin or doxorubicin-based regimens, have been investigated in selected cases, though their therapeutic efficacy remains uncertain. Intracavitary chemotherapy has shown promise in delaying effusion recurrence but has not demonstrated a clear survival advantage (12).

Overall, prognosis for pericardial mesothelioma is generally poor, and survival outcomes in dogs managed without chemotherapeutic interventions have historically been limited. Kerstetter et al. reported that only two dogs treated with surgery alone survived 4 and 9 months, and Stepien et al. described a median survival time of 60 days (range 15–300 days) following partial pericardiectomy, with the single longest survivor having received adjunctive chemotherapy (3, 13). Notably, multivariate analysis by Lajoinie et al. identified chemotherapy as the sole variable independently associated with survival in dogs with mesothelioma, further highlighting its recognized role in disease management (2). In this context, the present case report describes a canine patient who survived 628 days without any chemotherapeutic intervention, representing a markedly prolonged survival relative to previously reported outcomes. We propose that clinical stability may be achievable through proactive effusion management, subtotal pericardiectomy, and comprehensive supportive care alone, even in the absence of systemic chemotherapy. These findings may be of particular relevance in clinical settings where chemotherapy is financially or medically unfeasible, suggesting that this approach warrants consideration as a viable option prior to the decision for euthanasia.

## 2 Case description

**Figure 1:**
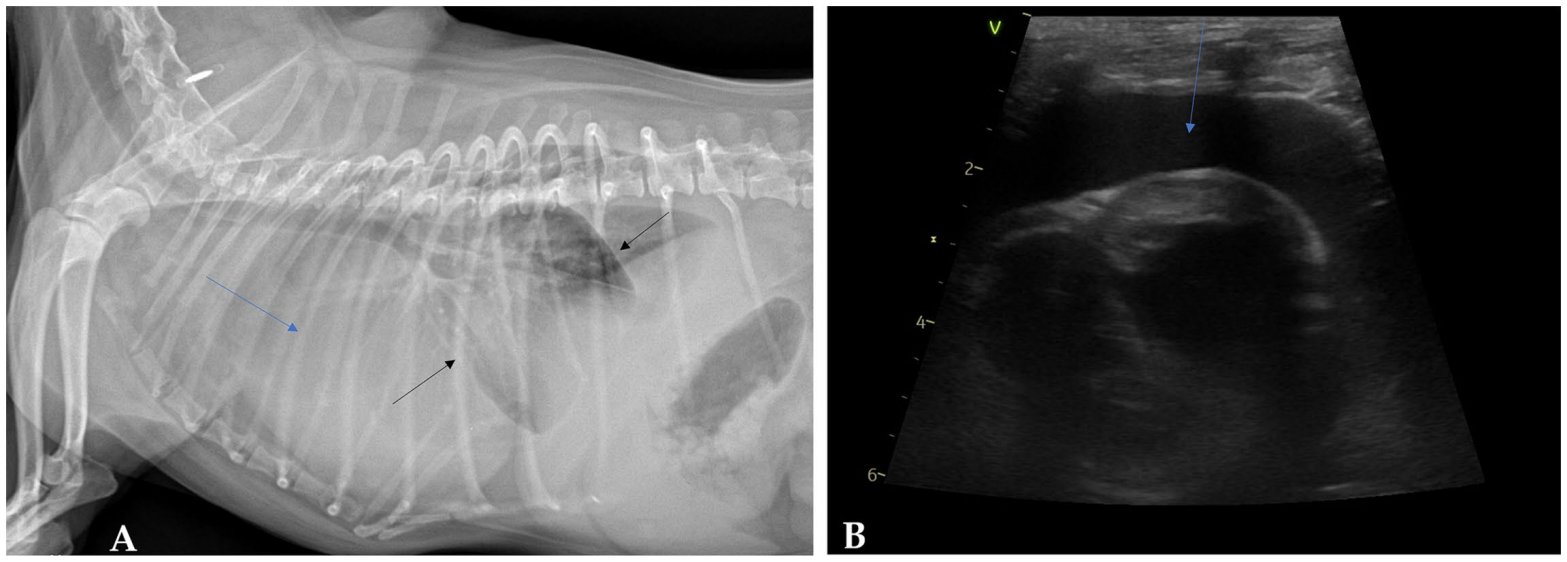
Diagnostic imaging findings at initial presentation. **(A)** Right lateral thoracic radiograph showing cardiomegaly (blue arrow) and evidence of pleural effusion (black arrows). **(B)** Echocardiographic image demonstrating moderate pericardial effusion (blue arrows).

A 7-year-old spayed female Pomeranian, weighing 4.58 kg, presented with an acute-onset respiratory distress, unproductive cough, and occasional gagging, persisting for approximately 24 h (Day 0). The patient maintained a normal appetite, though the owner noted concurrent lethargy. The dog lived indoors and no known exposure to toxic substances including smoke and asbestos. The dog had no prior history of systemic disease or surgical intervention except spaying, Physical examination revealed tachypnea with shallow respiratory patterns and muffled heart sounds on thoracic auscultation. Thoracic radiographs revealed marked cardiomegaly with a vertebral heart score (VHS) of 14.3 (reference interval [RI] 8.5–10.5) and partially obscured cardiac silhouette and overall increased radiopacity of the thorax, consistent with pleural fluid accumulation (Figure 1A) (14). Subsequent echocardiography confirmed a moderate pericardial effusion and mild tricuspid valve regurgitation, with no evidence of discrete intracardiac masses (Figure 1B).

Initial clinical pathology on Day 0 demonstrated a mixed respiratory and metabolic acidosis, evidenced by decreased pH of 7.19 (RI 7.35–7.42), markedly elevated PCO₂ of 63.9 mmHg (RI 35–45 mmHg), and reduced buffer base of −5.6 mmol/L (RI -4.5–0.3), while electrolytes remained within reference intervals. Hematologic analysis showed a hematocrit of 51% (RI 37–55%) and a hemoglobin concentration of 15.8 g/dL (RI 11.9–18.9 g/dL). Therapeutic intervention comprised ultrasound-guided pericardiocentesis and thoracocentesis, which removed 50 mL of pericardial fluid and 200 mL (Supplementary Figure 1) of pleural fluid, respectively, and led to rapid clinical improvement and resolution of respiratory distress. The patient was subsequently treated with pimobendan (Vetmedin, Boehringer Ingelheim, Germany; 0.3 mg/kg orally every 12 h), furosemide (Lasix, Handok, South Korea; 1 mg/kg orally every 12 h), and pentoxifylline (Trental, Sanofi, France; 20 mg/kg orally every 12 h). At the time of initial presentation, the echocardiographic assessment was necessarily limited in scope given the clinical urgency, and a complete characterization of cardiac function was deferred to the follow-up examination on Day 3. In this context of diagnostic uncertainty, pimobendan and furosemide were prescribed empirically based on the attending clinician’s concern for potential hemodynamic compromise secondary to pericardial tamponade, and pentoxifylline was administered for its hemorrheologic properties to improve microcirculatory flow in the setting of large-volume hemorrhagic bilateral effusions. Metronidazole (Flasinyl, HK inno.N, South Korea; 10 mg/kg orally every 12 h) was administered empirically due to the attending clinician’s concern for potential translocation of anaerobic bacteria following simultaneous pericardiocentesis and thoracocentesis in the context of large-volume bilateral effusions. Although thoracocentesis has been identified as an independent risk factor for pleural space infection in dogs and anaerobic bacteria are a common causative organism in canine pyothorax, we acknowledge that routine antimicrobial prophylaxis is not standardly recommended for these procedures under aseptic conditions, and this decision was based on individual clinical judgment (15).

A follow-up echocardiographic assessment on Day 3 provided further characterization of cardiac function. Left-sided cardiac dysfunction was excluded based on a normal left atrial size (LA/Ao 1.2;reference interval<1.6), absence of diastolic dysfunction and preserved left ventricular dimensions (E peak velocity 0.66 m/s;reference interval≤1.5 m/s, left ventricular internal dimension in diastole normalized 1.45;reference interval<1.7, left ventricular internal dimension in systole 14.71 mm; reference interval≤15 mm in a dog of 4 kg). Although myxomatous change of mitral valve was identified, peak mitral regurgitant jet pressure gradient of 143.8 mmHg was within the expected range for a normotensive dog, given that the normal mitral regurgitation velocity in dogs is approximately 5–6 m/s (16, 17). Right-sided congestive heart failure and pulmonary hypertension were excluded, as the tricuspid regurgitant pressure gradient of 17.55 mmHg (reference interval<30 mmHg) and pulmonic regurgitant pressure gradient of 13.5 mmHg (reference interval<19.5 mmHg) were both within normal limits (11). As no discrete intracardiac masses were identified and both left- and right-sided heart failure have been excluded idiopathic pericardial effusion was considered the primary working diagnosis. Nevertheless, infiltrative cardiac neoplasia including mesothelioma remained a differential diagnosis at this stage.

The patient remained stable without notable clinical signs for approximately 9 months then was hospitalized from Day 275 to Day 276 following recurrence of respiratory distress. A thoracic X-ray demonstrated persistent cardiomegaly (VHS 12.2), and respiratory parameters improved with pericardiocentesis and oxygen cage stabilization during this hospitalization period. On Day 276, echocardiographic evaluation confirmed recurrent pericardial fluid accumulation necessitating therapeutic drainage of 90 mL. Clinicopathologic evaluation showed mild respiratory acidosis (pH 7.33, PCO₂ 45.8 mmHg), elevated acute phase protein response (C-reactive protein 45 mg/L, RI 0–10 mg/L), and moderate thrombocytopenia (platelet count 148 × 10^9^/L, RI 150–500 × 10^9^/L). Serum biochemical analysis showed that hepatic and renal parameters were within reference intervals.

**Figure 2:**
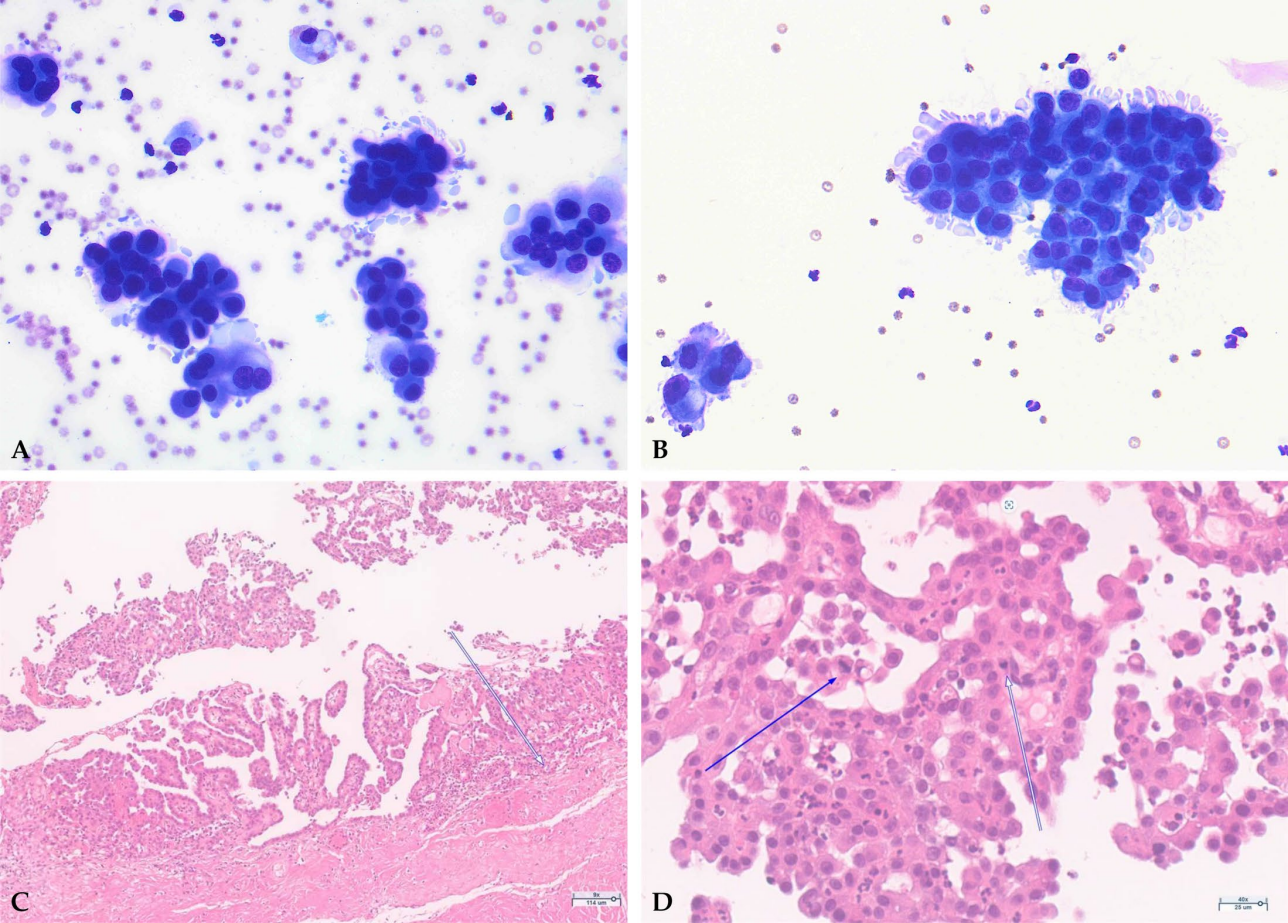
Histopathological and cytological findings. **(A)** Cytological analysis of pericardial fluid (x400) showing clusters of mesothelial cells with moderate anisokaryosis, fine chromatin, and eosinophilic cytoplasm. **(B)** Cytology of pleural effusion (x400) demonstrating mesothelial cells with nuclear pleomorphism and occasional nucleoli. **(C)** Histopathology of the pericardium (x100) showing papillary and glandular proliferations of cuboidal mesothelial cells supported by fibrovascular stroma. **(D)** High-power view of pericardial tissue (x400) showing neoplastic mesothelial cells with prominent nucleoli, moderate nuclear pleomorphism, and infiltration into the underlying connective tissue (arrows), confirming malignant pericardial mesothelioma.

Cytomorphologic assessment of the pericardial effusion revealed hemorrhagic effusion with mesothelial cell populations and total protein concentration of 4.0 g/dL, but no definitive neoplastic cellular criteria. Specifically, numerous erythrocytes admixed with mesothelial cells arranged in cohesive clusters or scattered individually. The mesothelial cells displayed relatively abundant basophilic cytoplasm and nuclei with moderate anisokaryosis (Figure 2). Definitive criteria for malignancy such as marked nuclear pleomorphism and atypical mitotic figures were not conclusively identified, underscoring the inherent limitation of cytology in distinguishing reactive from neoplastic mesothelial cells and the necessity for histopathological confirmation.

Between Days 276 and 520, the patient required progressively more frequent pericardiocentesis interventions, yielding drainage volumes ranging from 67 mL to 240 mL (Supplementary Figure 1). The intervals between procedures were shortened from monthly to biweekly. Adjunctive pharmacologic management comprised tranexamic acid (Transamin, Jeil Pharm, South Korea;10 mg/kg orally every 12 h), metronidazole (Flasinyl, HK inno.N, South Korea; 10 mg/kg orally every 12 h), pentoxifylline (Trental, Sanofi, France; 20 mg/kg orally every 12 h), and famotidine (Famotidine, Hanmi Pharm, South Korea; 0.5 mg/kg orally every 12 h), although effusion recurrence persisted. Coagulation parameters were assessed prior to initiation and found to be within reference intervals (PT 8.6 s [RI 6–10], aPTT 15.3 s [RI 10–25]), thereby excluding systemic coagulopathy. Given that a primary hemostatic defect was excluded, the hemorrhagic character of the recurrent effusion was attributed to local tumor-associated fibrinolytic activity at the serosal surface. Tranexamic acid was therefore administered to inhibit plasminogen activation at the local serosal level, with the aim of attenuating hemorrhagic effusion reaccumulation rather than correcting systemic coagulation deficits. We acknowledge that direct evidence for serosal fibrinolytic activity was not obtained in this case, and this rationale remains inferential.

**Figure 3:**
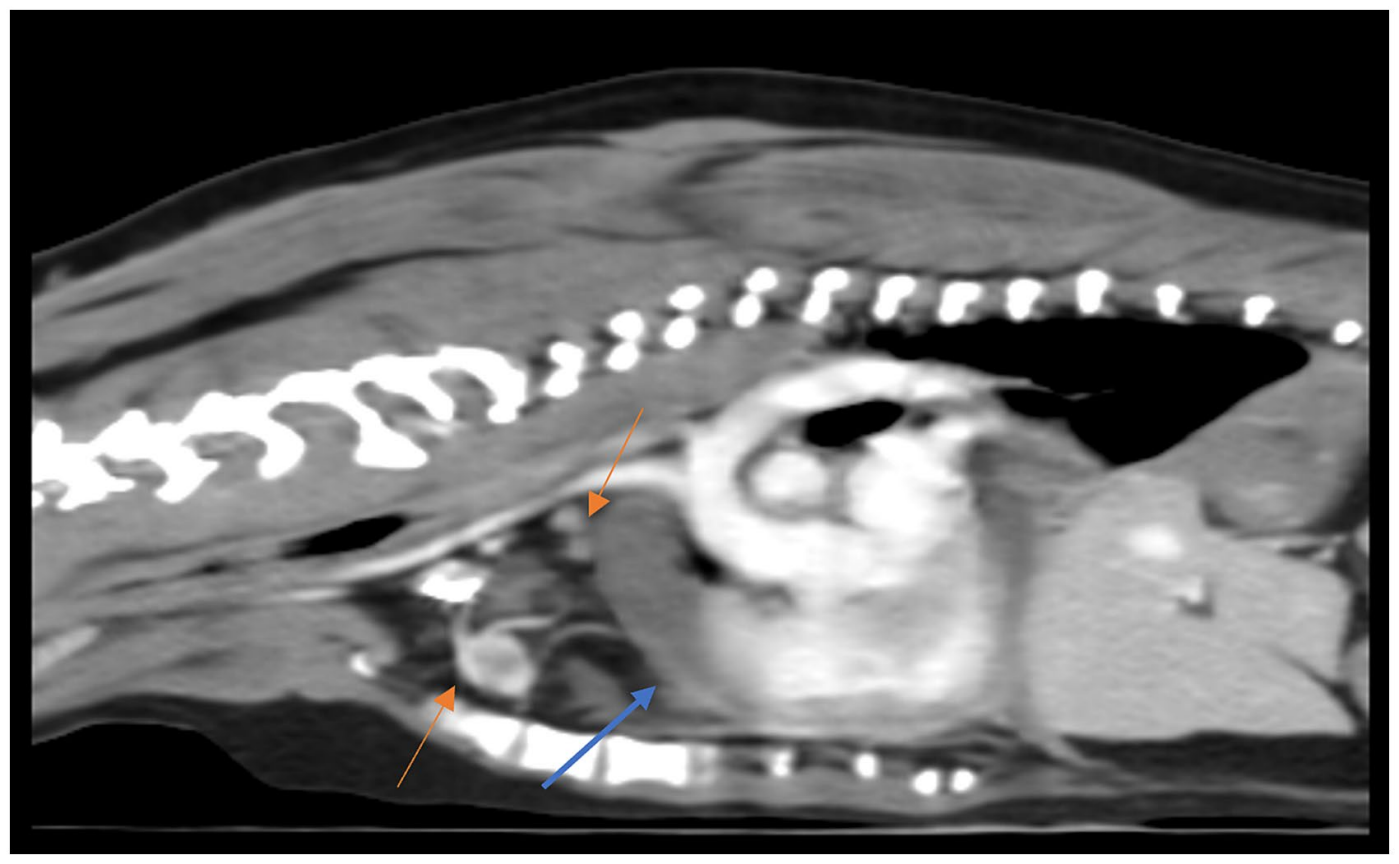
Computed tomography (CT) findings at diagnosis. CT images revealing pericardial thickening (blue arrow), regional lymphadenopathy (orange arrows), and nodular changes in the mediastinum, consistent with malignant pericardial mesothelioma.

CT scan was performed to investigate what was causing the persistent pericardial effusion and to inform about potential surgical options. CT imaging revealed free fluid within the thoracic cavity and pericardial sac, consistent with pleural and pericardial effusions. Heterogenous increased attenuation was observed in multiple pulmonary lobes. Mild enlargement of the sternal and cranial mediastinal lymph nodes was identified. Additionally, multiple atypical lesions with peripheral rim enhancement were scattered throughout the right thoracic musculature, accompanied by ground-glass opacity in the adjacent regions. In addition to pericardial wall thickening, these findings were considered consistent with a diffuse infiltrative neoplastic process, raising suspicion for mesothelioma (Figure 3). Concurrent cytological analysis of pericardial and pleural effusions was performed, while the surgically excised pericardium was examined histopathologically. Cytological analysis of the pericardial fluid (Figure 2A) identified clusters of mesothelial cells exhibiting moderate nuclear anisokaryosis, finely granular chromatin, and abundant eosinophilic cytoplasm. Scattered erythrophagocytic macrophages and mild neutrophilic infiltration were also observed. Concurrent cytological analysis of the pleural effusion revealed a serosanguinous effusion in which mesothelial cells were the predominant cell population, present in clusters of varying sizes or occasionally as individual cells. Individual cells exhibited basophilic cytoplasm of variable abundance with occasional cytoplasmic blebbing at the cell margins. Nuclei were round to oval with a high degree of anisokaryosis, fine chromatin, and a single prominent nucleolus, accompanied by mild neutrophilic pleocytosis. Although atypical mesothelial cells raised suspicion for a neoplastic process, definitive cytological criteria for malignancy and invasive behavior were not conclusively observed, underscoring the need for histopathological confirmation.

A subtotal pericardiectomy was performed via a right lateral thoracotomy approach to facilitate continuous drainage of the pericardial effusion and to obtain diagnostic tissue. The pericardium was resected to the extent technically feasible. Histopathological examination of the excised pericardium was performed across six tissue sections. At low magnification, the pericardial tissue demonstrated moderate to severe papillary arboriform and gland-like projections extending into the inner surface. At intermediate magnification, these papillary structures were composed of fibrovascular connective tissue stalks lined by single or multiple layers of cuboidal basophilic mesothelial cells. At high magnification, the neoplastic cells exhibited large oval nuclei with prominent nucleoli and abundant eosinophilic cytoplasm. In deeper regions, acinar and glandular structures resembling adenoma or adenocarcinoma were identified, representing invaginated growth of mesothelial cells. Mitotic figures were observed at a frequency of 0–1 or more per high-power field, and neoplastic cells demonstrated clear invasive tendency into the underlying connective tissue (Figures 2C,D, arrows). These histopathological features were considered inconsistent with reactive mesothelial hyperplasia, in which mesothelial proliferation typically remains superficial, lacks true stromal invasion, and does not form complex papillary or glandular structures. The constellation of findings was therefore diagnostic of malignant pericardial mesothelioma. However, the owner declined chemotherapy due to concerns about cost and potential side effects.

**Figure 4:**
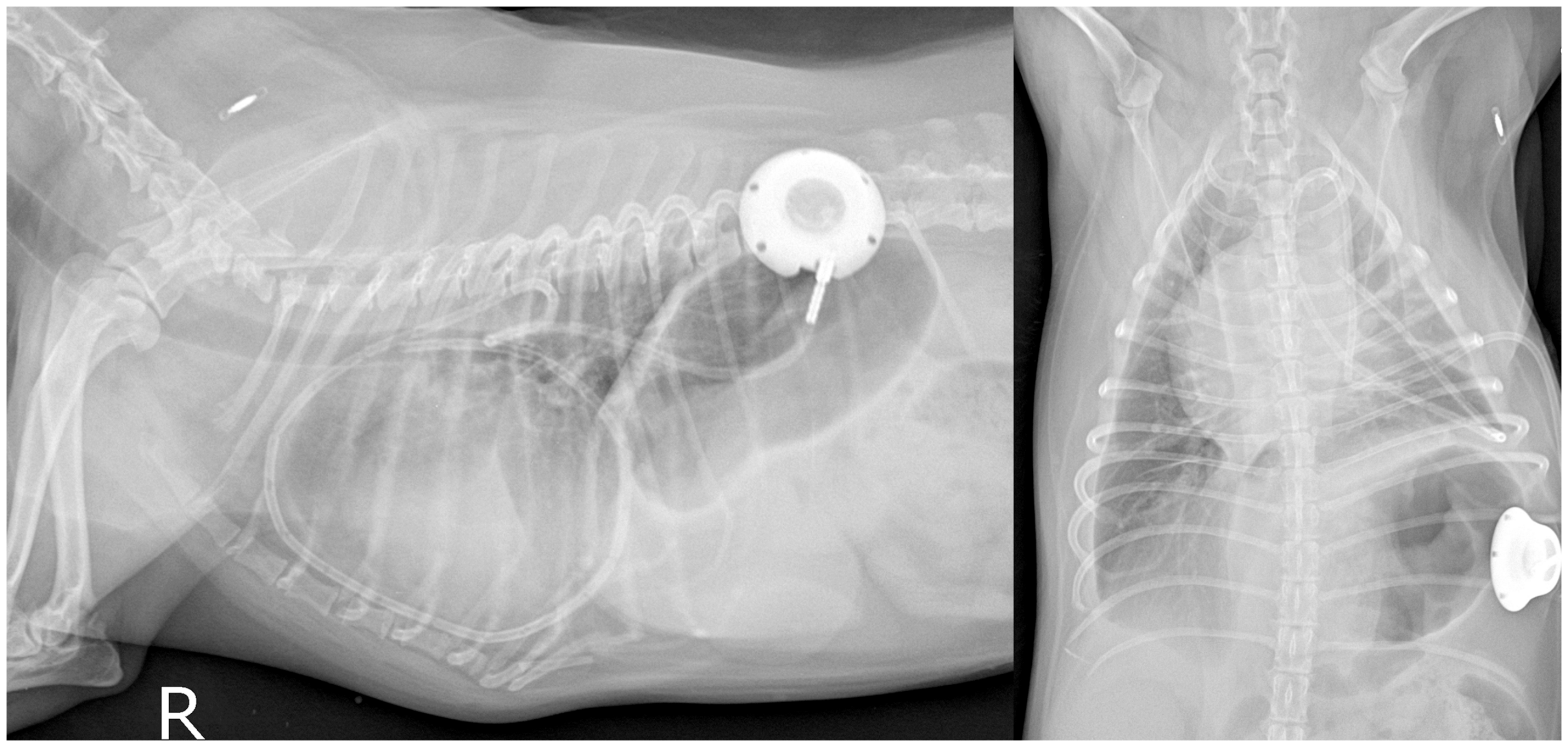
Postoperative thoracic radiograph demonstrating the indwelling thoracic drainage port implanted in the left thoracic cavity for long-term management.

Despite undergoing subtotal pericardiectomy and concurrent placement of a thoracic drainage port (Figure 4), the pleural effusion recurred progressively from Day 540, necessitating regular drainage via the previously implanted thoracic port. Drainage volumes per session ranged from 70 to 240 mL, with intervals initially between seven and 10 days, which progressively shortened over time (Supplementary Figure 1). Cytological analysis of the drained pleural fluid revealed moderate cellularity (white blood cell (WBC) count of 10.21 × 10^3^/μL) and elevated protein concentration (4.0 g/dL), primarily comprising atypical mesothelial cells with moderate nuclear pleomorphism, fine chromatin, and abundant eosinophilic cytoplasm (Figure 2B).

No major procedure-related or drug-related adverse events were observed throughout the follow-up period. However, because of ongoing clinical deterioration including progressively increasing respiratory effort and increased frequency and volume of thoracic fluid drainage, prednisolone (Korea Pharma, South Korea) was initiated on Day 620 at a dose of 0.5 mg/kg orally daily per once, which was subsequently increased to 1 mg/kg orally twice daily, as palliative anti-inflammatory therapy aimed at reducing the rate of pleural effusion accumulation and improving the patient’s comfort and appetite in the context of progressive clinical deterioration. The initial response to prednisolone therapy was favorable, temporarily extending intervals between fluid drainage procedures. However, by Day 626, thoracic drainage was required every 1–2 days, with fluid removal volumes ranging from 80 to 120 mL (Supplementary Figure 1). The patient’s body weight fluctuated between 5.88 kg and 6.02 kg during this period. Despite intensive supportive care and repeated thoracic fluid drainage, the patient’s clinical condition progressively worsened. On Day 627, scheduled thoracic fluid drainage was deferred due to the patient’s severely compromised condition, and the patient subsequently succumbed to respiratory arrest on Day 628. The overall survival was approximately 21 months (628 days) following the initial presentation of pericardial effusion.

## 3 Discussion

This case report describes a rare instance of prolonged survival (21 months) in a dog diagnosed with malignant pericardial mesothelioma, managed without chemotherapy. This outcome may suggest the potential importance of pericardial and pleural fluid management, subtotal pericardiectomy, and supportive therapies in optimizing survival and maintaining quality of life in cases where chemotherapy is not feasible due to medical or practical limitations, such as poor patient tolerance or financial constraints. Previous studies have documented median survival times (MST) for canine malignant pericardial mesothelioma ranging from 74 to 366 days, depending on the treatment approach (1, 2). While surgical intervention and adjunctive therapies have been associated with extended survival, overall prognosis remains guarded, with only 22% of affected dogs surviving beyond 1 year (1, 8). The 21-month survival observed in this case may suggest the potential benefits of proactive medical and surgical management in prolonging survival and improving quality of life.

Establishing a definitive diagnosis was initially challenging because of the non-specific clinical presentation and inconclusive cytological findings. Thoracic radiographs, echocardiography, and CT failed to identify discrete mass lesions, which is consistent with the known infiltrative growth pattern of mesothelioma, wherein the neoplasm diffusely involves serosal surfaces rather than forming well-defined nodular masses (1, 10). Cytological evaluation of pericardial and pleural fluid (Figures 2A,B) revealed atypical mesothelial cells with anisokaryosis and occasional nucleoli, but definitive malignant characteristics were absent.

Histopathological analysis of the excised pericardium confirmed malignant pericardial mesothelioma, characterized by papillary and glandular proliferations of mesothelial cells displaying moderate nuclear pleomorphism, abundant eosinophilic cytoplasm, prominent nucleoli, and occasional mitotic figures, with invasion into adjacent connective tissues (Figures 2C,D), consistent with previously described histologic features of canine pericardial mesothelioma (1, 12, 18). An important diagnostic consideration in this case was the histopathological distinction between malignant mesothelioma and severe reactive mesothelial hyperplasia, which can occur in dogs with idiopathic pericardial effusion and may exhibit overlapping cytological and even histological features. In the present case, several histopathological features supported the diagnosis of malignant mesothelioma over reactive pericarditis: the presence of complex papillary arboriform and glandular structures, multilayered mesothelial cell proliferation with marked nuclear atypia and prominent nucleoli, mitotic figures, and most critically, unequivocal invasion of neoplastic cells into the underlying connective tissue stroma. Stromal invasion is recognized as the most reliable histopathological criterion for distinguishing malignant mesothelioma from reactive mesothelial hyperplasia, as reactive processes do not demonstrate true tissue invasion (19). Although cytological analysis of pericardial and pleural fluid identified atypical mesothelial cells, the findings lacked definitive features of malignancy. This underscores the limited sensitivity and specificity of cytology in detecting pericardial mesothelioma and highlights the importance of histopathological evaluation for definitive diagnosis (1, 2, 12, 20).

In this case, systemic chemotherapy was not undertaken due to the owner’s concerns regarding cost and potential side effects; instead, therapeutic interventions included subtotal pericardiectomy and concurrent thoracic port placement. Subtotal pericardiectomy alleviated recurrent cardiac tamponade, a common complication of pericardial mesothelioma, thereby improving cardiac function and clinical status (5). Additionally, the thoracic port facilitated the long-term management of recurrent pleural effusion, contributing significantly to the patient’s clinical stability and quality of life.

Several prognostic factors potentially contributed to the extended survival time observed in this case. Early recognition of clinical signs and immediate therapeutic interventions likely prevented severe cardiac dysfunction and secondary organ impairment. Additionally, a structured follow-up protocol involving consistent clinical evaluations and fluid management facilitated timely interventions that helped maintain clinical stability. Another essential factor was the high level of owner compliance with the therapeutic regimen, enabling effective long-term management. Finally, the patient’s relatively young age at diagnosis (7 years) and lack of significant concurrent conditions likely improved tolerance to repeated invasive procedures and physiological resilience, further supporting prolonged survival.

The progression from primary pericardial involvement to subsequent pleural effusion reflects a characteristic pattern of malignant mesothelioma dissemination. Typically, mesothelioma originates as a diffuse neoplastic transformation of mesothelial cells lining the pericardium, spreading insidiously into adjacent structures rather than forming clearly defined masses (10, 21, 22). This infiltrative growth pattern accounts for the initial diagnostic challenges and the absence of discrete masses on imaging. The progression from predominantly pericardial to pleural effusion following pericardiectomy may reflect continued neoplastic dissemination to the pleural mesothelium, which is characteristic of malignant mesothelioma (23). While it is possible that removal of the pericardial barrier may have facilitated preferential fluid accumulation in the pleural space by reducing local resistance, we acknowledge that this interpretation is speculative and is not supported by direct evidence in the present single case report (23, 24). Regardless of the underlying mechanism, this pattern of disease progression highlights the importance of viewing the pericardial and pleural spaces as interconnected compartments in dogs with mesothelioma, with implications for both long-term monitoring strategies and therapeutic planning (25).

In veterinary oncology, maintenance of body weight has been recognized as a positive prognostic indicator, as cancer cachexia and progressive weight loss are associated with reduced survival and diminished quality of life (26–28). In the present case, a gradual increase in body weight was observed throughout the clinical course, from 4.58 kg to 6.02 kg, which may be interpreted as a reflection of sustained clinical stability over the prolonged survival period. This weight maintenance may further reflect effective control of disease-associated clinical signs including nausea, discomfort, and respiratory compromise (29). While formal body condition scoring and dietary assessments were not performed, limiting definitive conclusions, the observed weight trajectory in this patient is consistent with the favorable prognostic trend reported in the oncological literature (30).

This single-case report has several limitations, including its retrospective design and the inherent constraints of individual case documentation. Additionally, as chemotherapeutic intervention was not implemented, definitive comparisons between chemotherapy and supportive management cannot be established. Also, immunohistochemical analysis was not performed due to resource constraints. While IHC markers including calretinin, WT1, CK5/6, and D2-40 may support a diagnosis of malignant mesothelioma, no marker conclusively defines the mesothelial lineage, and approximately 30% of mesotheliomas exhibit a null phenotype with negative staining for all markers (10). The diagnosis therefore relied on morphologic assessment in conjunction with clinical and imaging findings, consistent with current diagnostic practice. Further investigation is necessary to evaluate the comparative effectiveness of various therapeutic strategies, including chemotherapy, surgical interventions, and palliative care, in canine pericardial mesothelioma. Multi-institutional studies examining survival outcomes across different treatment approaches would provide valuable data to enhance prognostic accuracy and develop evidence-based clinical guidelines.

Clinical recommendations derived from this case include a sequential diagnostic protocol beginning with non-invasive or minimally invasive techniques, such as thoracic imaging, cardiac ultrasonography, and fluid analysis. When initial diagnostic results are inconclusive, other imaging modalities (e.g., CT) and tissue sampling should be pursued for accurate diagnosis and treatment planning. Monitoring frequency should be adjusted according to effusion recurrence patterns. For this patient, evaluations every 2 weeks were initially adequate, with subsequent transition to more frequent assessments (weekly, or more often) as their condition progressed. The implementation of individualized monitoring protocols, adjusted according to disease progression and effusion recurrence patterns, may facilitate an optimal balance between rigorous clinical surveillance and sustained owner compliance. Prompt consideration of subtotal pericardiectomy is indicated for cases with recurrent effusion unresponsive to medical therapy, as this intervention both reduces cardiac tamponade risk and provides diagnostic tissue specimens. In patients experiencing rapid pleural fluid reaccumulation, placing an indwelling thoracic port should be considered, as it facilitates efficient fluid removal while reducing patient anxiety and the need for sedation or anesthesia.

This case described a dog with pericardial mesothelioma that survived for 21 months without chemotherapy. The treatment plan involved sequential effusion drainage, subtotal pericardiectomy, and thoracic port implantation. Our findings suggest that survival may be prolonged without chemotherapy, which is of particular clinical relevance in scenarios where owners decline chemotherapy or where systemic treatment is deemed medically unsuitable. Further validation of these observations through larger case series would allow refinement of protocols.

## Data Availability

The original contributions presented in the study are included in the article/Supplementary material, further inquiries can be directed to the corresponding author.

## Supplementary material

SUPPLEMENTARY FIGURE 1 Changes in pericardial and thoracic fluid drainage and body weight over time in a dog with malignant pericardial mesothelioma. Pericardial and thoracic fluid drainage volumes (mL) and body weight (kg) were monitored throughout the disease course. “Day” indicates time since diagnosis. Fluid drainage (mL) reflects pericardial and thoracic centesis volumes.
